# A Case Report and Literature Review of Death by Potassium Chloride: Is it a Forensic Medicine Enigma?

**DOI:** 10.7759/cureus.57776

**Published:** 2024-04-07

**Authors:** Lucia Tarda, Matteo Antonio Sacco, Saverio Gualtieri, Wandamaria Mazzuca, Isabella Aquila

**Affiliations:** 1 Institute of Legal Medicine, Department of Medical and Surgical Sciences, Magna Graecia University of Catanzaro, Catanzaro, ITA

**Keywords:** potassium chloride poisoning, potassium, suicide, forensic sciences, death, autopsy

## Abstract

The diagnosis of death from potassium chloride (KCl) injection is complex because it is a natural ion, so it is very difficult to trace the exogenous administration of drugs containing potassium. In these cases, it is important to perform a careful cadaver inspection, with the collection of circumstantial data on the scene, and proper autopsy investigations, in order to avoid errors regarding the reconstruction of the cause of death. The aim of this paper is to propose a forensic protocol, including evidence collected from the literature, that could help the medical examiner to perform the correct diagnosis, and also encourage control measures to avoid similar events, especially in nosocomial environments. This is important, especially for health professionals who can easily access drugs containing potassium. For this reason, we need to strengthen control measures of these drugs by proposing a double check in cases of pharmacological use, specifying the volume taken with a double authorization signature and the reasons for the withdrawal.

## Introduction

Potassium chloride (KCl) is a chemical compound, formed by the union of a potassium atom with one of chlorine that, when it is introduced into the body, dissociates into the two elements [1]. This compound is used in cases of deficiency of potassium ion (K^+^) levels, and in some diseases such as diabetic ketoacidosis or hyperaldosteronism [2]. It is also an essential, ubiquitous ion, with functions related to muscle contraction, pulse transmission, and acid-base balance, so altered levels of K^+^ can generate numerous pathological consequences, sometimes even fatal.

A strict control over the administration and storage of KCl is required and it is usually carried out only by experienced health professionals. In fact, high doses of KCl may determine adverse effects such as vomiting, gastrointestinal disorders, neuromuscular disorders, paresthesia, flaccid paralysis, mental confusion, arrhythmias, and elongation of QRS, and even the fatal condition of sudden cardiac death. Death from acute potassium intoxication generally occurs due to the onset of acutely fatal arrhythmic complications. 

The literature shows that the diagnosis of death from KCl is complex because it is an endogenous ion, so it is difficult to trace an exogenous administration. The literature suggests the difficulty in evaluating these cases is also due to the phenomena of post-fatal hemolysis which could lead to an increase in potassium blood levels [3-5]. Some authors have suggested the difficulties related to autopsy findings alone and the need to integrate autopsy data with crime scene data and with advanced laboratory investigations, such as scanning electron microscopy-energy dispersive X-ray analysis (SEM-EDX) [3-5]. There are few cases reported in the literature of murders/suicides carried out using K^+^ [3-5].

In this paper, we want to describe a case of suicide carried out by using KCl and also draw up a protocol about the investigations necessary to formulate the diagnosis of death by exogenous injection of KCl, that could be useful for forensic purposes.

## Case presentation

Case history

A 40-year-old nurse was found dead in his home, in a prone position, with intense cyanosis and a band-aid on his left arm. Analysis of the scene revealed the presence of a syringe placed on the table of the room soaked in blood; moreover, an empty "potassium chloride" bottle bearing the words "fatal if not diluted" was found. The autopsy was ordered by the Judicial Authority in order to clarify the cause and manner of death.

Crime scene investigation

The scene investigation was carried out, with the collection of circumstantial data, external examination of the body, and autopsy. Samples were taken of the skin crease of the left arm, where the needle mark was present, and fragments of organs. Samples of biological fluids (blood, vitreous humor, urine) were collected. A qualitative and quantitative toxicological analysis was carried out, characterized by a screening performed on blood after extraction with trichloroacetic acid and acetonitrile, in an immunoenzymatic method, for alcohol, drugs, and psychopharmaceuticals. Solid phase microextraction (SPME) was performed over head space (HS-SPME), with a gas chromatograph coupled to a flame ionization detector (FID). The syringe was seized, as was the potassium vial at the scene. Investigators evaluated the origin of the vial by analyzing the codes on the vial in order to identify how the victim obtained the drug.

Analysis of vial and syringe found on the scene

**Figure 1:**
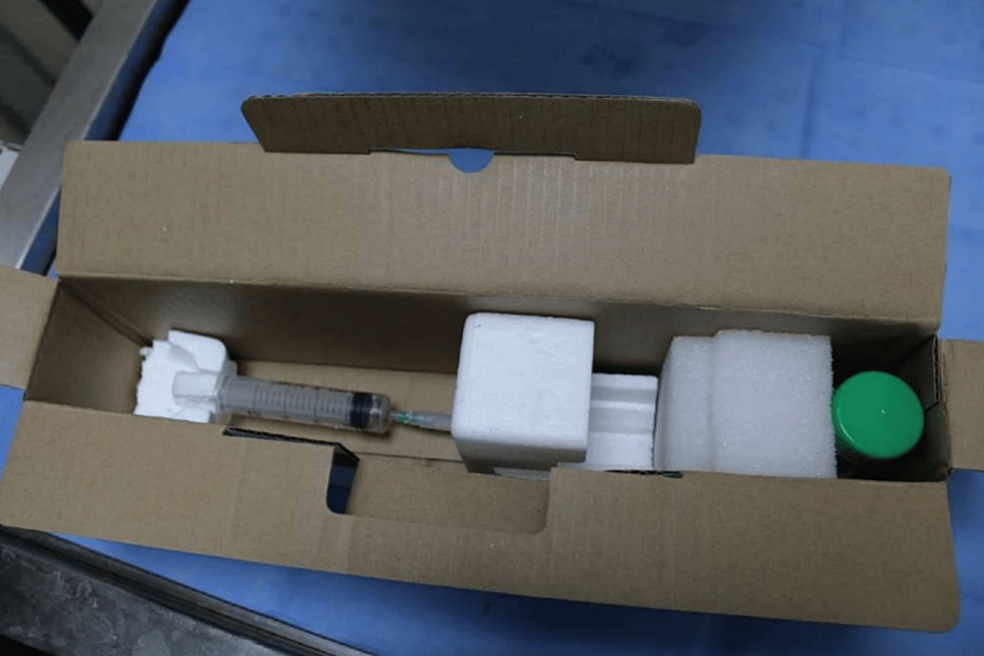
The syringe used for potassium injection

From the analysis of the vial seized during the scene investigation, the presence of KCl was found. The substance was compared with that inside the syringe, found near the body on the scene (Figure 1).

The comparison showed the match of the two substances proving that the substance in the vial corresponded to the substance in the syringe used by the man. In addition, the vial presented on the outer surface the name with the product identification code, and the identification number corresponded, in turn, to a box in the hospital where the victim worked.

Autopsy findings

**Figure 2:**
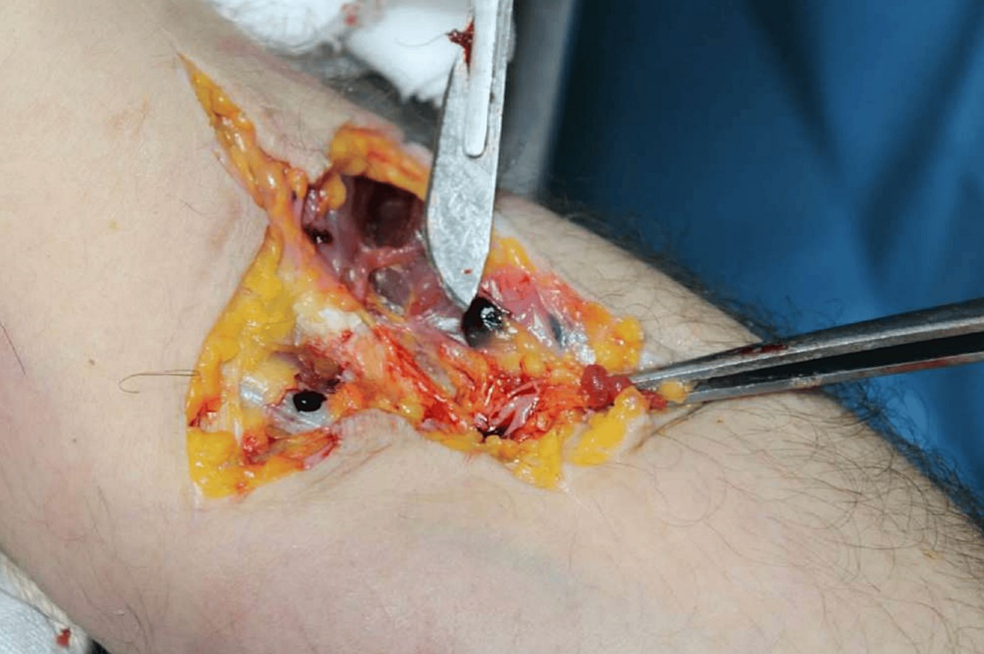
Incision of the needle mark on the left arm showing hemorrhagic infiltrate of subcutaneous and muscular tissues

External examination of the body showed rigor mortis on the upper and lower limbs, sub-conjunctival hyperemia, vomiting from the buccal orifice, small cutaneous petechiae spread on the anterior region of the neck and on the right shoulder, and bruising at the needle mark on the left arm. In this region, a skin incision was made showing hemorrhagic infiltration of the subcutaneous and muscular tissues (Figure 2).

Internal examination revealed intense blood congestion of organs such as the lungs, spleen, kidneys, and thyroid, accentuated vascularization, especially in the small intestine. The heart showed whitish spots circumscribed on the anterior and posterior epicardial surface with a great number of clots; the stomach presented partially digested food content, and hyperemia of the gastric mucosa; alimentary regurgitation in the glottis was evident. A histological examination was performed and it proved hemorrhagic infiltration at the injection site, confirming blood congestion of the organs and signs of ventricular fibrillation. Also, the presence of signs of alimentary regurgitation suggested gastrointestinal symptoms before death, according to the side effects of hyperkalemia. No injuries were found on the brain.

Toxicology findings

The toxicological investigation on biological fluids excluded the presence of drugs or psychotropic drugs. Alcohol was found in the blood at moderate levels (0.80 g/L). Potassium concentration was 90 mM in femoral blood.

According to the crime scene, autopsy, histological, and toxicological findings, the cause of death was acute cardiorespiratory failure secondary to acute asphyxia with ventricular fibrillation attributable to intravenous injection of KCl.

## Discussion

The first phase of the investigation was the scene analysis and it was fundamental for the discovery of circumstantial evidence such as the syringe and the vial containing KCl. The investigations proved that the vial was taken from the hospital where the man worked as a nurse. The literature often mentions the importance of circumstantial evidence in similar cases [4-7]. For this reason, the crime scene investigation must be carried out by specialists in forensic medicine to allow the proper recognition of traces or evidence on the scene and on the body suggesting the use of exogenous substances. So, if not recognized immediately, all necessary investigations may not be carried out, with the risk of errors in the assessment of the case.

In the reported case, the external examination showed three elements that helped to analyze the case. The first element was the finding of vomiting at the mouth. It is a known fact that hyperpotassemia has side effects and among the most common are nausea, vomiting, abdominal disorders, and diarrhea. A second element was the acupuncture mark on the left arm. To assess the vitality of the injury, a skin incision was performed showing hemorrhagic infiltrate of the subcutaneous and muscle tissues. The bruised skin region was taken on the left arm where the acupuncture mark was present, during the autopsy, with the possibility of further toxicological investigations. In addition, the skin sample on the same region on the right arm was taken as "control". The third element was the absence of other injuries thus excluding a homicide. 

It would have been very difficult to come to this conclusion if both the scene investigation and autopsy had not been carried out. The autopsy showed intense blood congestion in the organs. Blood congestion has been found in the literature regarding exogenous injection death of KCl [5]. Zhang et al. reported pulmonary congestion and signs of dotted hemorrhage scattered in the myocardium in such a case [5]. Coulibaly et al. described a case of a fetus in the 36th week that was aborted with umbilical administration of 10% KCl, in which "deposits of whitish crystals" were observed during histopathological analyses of the endocardium, epicardium, myocardium, spleen, kidneys, and liver [7]. 

Literature data states that in death from KCl, the concentration of potassium in the central blood is significantly higher than the peripheral blood suggesting a post-mortem distribution [6]. In the current case, toxicological analysis proved the subject was in a state of lucidity at the time of death, since only moderate levels of alcohol were found. Toxicological investigations proved the presence of KCl in the syringe found on the scene. 

The case reported shows the importance of drug control in nosocomial environments, especially for healthcare providers who have the responsibility of the chain of custody, in order to reduce the incidence of drug theft. Therefore, proper methods of prevention should be adopted, such as the keeping of drugs under lock and key in the ward, the requirement of the presence of a health supervisor for the withdrawal of drugs, and the presence of double-signed registers indicating the volume of the drug taken, the patient for which it is to be used, and the reasons for the request.

## Conclusions

The case presented and the literature review propose a forensic protocol to guide the medical examiner in the analysis of similar cases of intoxications from KCl. The protocol includes immediate scene investigation, autopsy with collection of post-mortem biological fluids, and acupuncture mark analysis. A qualitative and quantitative toxicological analysis of post-mortem biological fluids and syringes used must be performed. Adequate surveillance is necessary, especially in hospital or pharmacy settings, where such products are more easily available.
